# "The Hospital" Nursing Mirror

**Published:** 1901-09-28

**Authors:** 


### "The Hospital" Nursing Mirror.

Being the Nursing Section of "The Hospital."

[Contributions for this Section of "The Hospital" should be addressed to the Editor, "The Hospital" Nursing Mirror, 28 & 29 Southampton Street, Strand, London, W.C.]

### Notes on News from the Nursing World.

### ARMY SISTERS AND ORDERLIES.

"What do army nursing sisters think about the need for a nursing department of the Royal Army Medical Corps?" asks the medical officer in charge of the embarkation duties at Southampton. They are not, we think, as he hints in favour of "sweeping the orderly off the face of the earth." But we are quite sure that they will endorse his opinion that "a loafer who has been hanging round the public house until the recruiting sergeant picks him up, cannot be turned into a good nurse all at once," if at all. The idea of some people, to which he refers, "that a detachment of orderlies should be turned into the wards of the London hospitals," is, of course, absurd. Neither sisters, nor nurses, are likely to be afforded the opportunity of showing whether they like or dislike such an outrage. The urgent importance of a reform of the present system, under which it has been proved that a number of more or less incapable orderlies are given employment, has already been pointed out in our columns.

### A STRANGE STORY.

A matron told our representative a strange story the other day. It was about a nurse who had volunteered for the front, and who was found weeping when the batch of nurses arrived at the base hospital. "What on earth is the matter?" the other nurses asked. "Oh," she sobbed, "shall I have to nurse men?" "Why, of course! Where were you trained?" "Oh, I was only trained at Queen Charlotte's, and I thought perhaps the soldiers' wives might want me!" We have heard some odd things about the experience, or non-experience, of some of the nurses who have been allowed to tend invalid soldiers in South Africa, but this sounds exceedingly like a severe satire.

### FROM SOUTH AFRICA.

The following members of the Army Nursing Reserve have arrived at Southampton from South Africa, on board the s.s. *Tagus*:—E. Fry, C. Terry, B. Blooks, and M. H. Franklin (all returning on expiration of leave), C. Brown, R. Bullock, and F. Low (invalids). The *Hawarden Castle*, which has on board nursing sisters W. M. Pooler and G. Chinnery, is due at Southampton on October 6.

### THE NEW MATRON OF LAMBETH INFIRMARY.

An excellent appointment has been made by the Lambeth Board of Guardians to the vacancy caused by the retirement of Miss Griffiths. The new matron of Lambeth Infirmary, one of the most important poor-law institutions in London, is Miss Emma Mary Byles, a lady whose training and experience abundantly justify her selection. Trained at Addenbrooke's Hospital, Cambridge, and St. Thomas's, London, Miss Byles afterwards went back to Cambridge, and having been engaged in private nursing for a year returned to Addenbrooke's as night superintendent. In 1893 she became ward sister, and occupied this post until 1899, when she was appointed assistant matron at St. Saviour's Infirmary, East Dulwich. She now leaves East Dulwich for Lambeth, where she will find ample scope for her abilities.

### A HUNDRED AND FOURTEEN APPLICATIONS FOR A POST OF MATRON.

The Committee of the West Cornwall Miners' and Women's Hospital were greatly astonished to receive no less than 114 applications for the appointment of matron, held by Miss Lawson, who has recently resigned. They found it most difficult to determine which of the 114 were most desirable, a very large percentage having thoroughly good references as to qualifications. After selecting those only who had previously acted as matrons the Committee felt that the matter was in some measure simplified, but still it was far from easy to choose four out of the many thoroughly competent ladies to be interviewed. On Wednesday, last week, after seeing the selected four candidates 16 of the committee recorded their votes for Miss Margaret Brewster, who was trained at the Royal Southern Hospital, Liverpool, and has held several important posts, including that of matron of the Borough Hospital, Ashton-under-Lyne, and 15 for a Cornish lady trained at the "London," the former securing the appointment. During the last 30 years there have been four matrons of the hospital, two of whom died after lengthened terms of office, one got married, and the fourth resigned, creating the vacancy which has just been filled.

### IMPROVEMENTS AT CHARING CROSS HOSPITAL.

The sisters and nurses returned to their duties at Charing Cross Hospital on the 19th inst., after an enforced holiday of two months, while building operations were carried out. "You can't feed patients when you have no kitchens," the matron said to our representative; "and if there are no patients there is nothing for the nurses to do." This is why they have all had their holiday at once instead of in detachments as usual; but the new kitchens are now complete, and the patients were received on the 21st, two days after the return of the staff. "Sister Victoria" was the first to appear, and the rest followed during the evening, to find charming little annexes to the wards, for their special convenience. These are small white-tiled lavatories, in which Doulton's sinks are provided for washing all the vessels used in the wards; a new patients' ward entrance has also been added; and both have hot and cold water and electric light laid on. The matron has been at the hospital during the progress of the alterations. The next thing required is a new nurses' home, and this will probably be commenced almost at once. It is expected that the work will occupy a considerable time. The home will be in the King William Street corner of the hospital's property.

### THREATENED COLLAPSE OF A SCOTTISH NURSING ASSOCIATION.

At the annual meeting of the North Argyle Nursing Association, Lady Victoria Campbell in the chair, the secretary told a doleful tale. Although the six nurses belonging to the association had discharged arduous duties in various parts of Mull and Ardnamurchan, one being also resident in the Island of Tiree, and during the twelve months had attended nearly 300 patients, the income of the organisation was only £269, as against an expenditure of £332. The secretary proceeded to say that "for several years" the expenditure had exceeded the income, with the result that the funds on hand had fallen so low that he had not sufficient left to carry on the work of the association for three months. In view of this state of matters, he added that he was instructed to print and circulate a statement of affairs, appealing to those interested in the nursing of the sick poor to contribute sufficient to bring the reserve fund up to its proper limit. It was decided that in the event of the response to this appeal proving inadequate, the secretary should be asked to call a general meeting, with a view to winding up the affairs of the association. This is a very discreditable state of affairs, and we hope that the people who know the value of the work done by the nurses of the North Argyle Association in the past will put their shoulder to the wheel, and not only stay the threatened collapse, but obtain sufficient support to place it on a sound financial basis.

### THE MATRON OF ST. BARTHOLOMEW'S.

Miss Isla Stewart, matron of St. Bartholomew's Hospital, is taking her holiday in America, where she will make an extended tour, of course including Buffalo. She intends visiting several hospitals, and will no doubt bring back some hints which will be found helpful to the nursing staff at St. Bartholomew's.

### THE NORTH INDIA SCHOOL OF MEDICINE FUND

An appeal is made on behalf of the North India School of Medicine for Christian Women at Ludhiana, Punjab. It is six years ago since this, the only medical school for women in India where the students are taught by women, where they can study apart from the Hindu and Mahomedan male students, and where they can receive their clinical teaching in a hospital for women and children, was opened. The hospital is now recognised by the Government as a medical school; 11 different societies have sent students for training; 8 medical students, 5 compounders, 8 nurses, and 6 midwives have completed their course of study at the school, and are now engaged in medical mission work in connection with 7 different societies; 16 medical students, 16 nurses, and 2 compounders are still in training; and while many applications are sent in to the hospital from trained workers, the reports received about most of the trained students are very encouraging. The University of Lahore is willing to affiliate the school as soon as three more members are added to the teaching staff, and good laboratories for anatomy, chemistry, physiology, and bacteriology have been provided. For this purpose, to provide an operating theatre and to supply the outfit and passage of the extra members of the staff, a sum of £5,000 is needed. Subscriptions may be sent to Lady Macnaghten, 198 Queen's Gate, S.W., or Miss E. Brown, M.D., 121 St. James's Road, Croydon.

### A SAD STIMULUS.

It used to be said of one of the English railway companies that if only a bishop would get killed on the line the directors would take steps to prevent further accidents. The death of a nurse from typhoid in the hospital attached to the Belfast Workhouse seems to have put the Belfast Board of Guardians to shame, and forced from them the acknowledgment that improvements in the conditions of nursing are really needed. In moving a vote of condolence with the relatives of the late Nurse Preston, one of the guardians said that the event should "be a stimulus to them to endeavour, if possible, to avoid an occurrence of that nature in the future." He then expressed the opinion that "the whole nursing staff of the house did require very considerable improvement," and "while there had been a sufficient conflict of opinion on the matter to prevent the Board from being unanimous in the method of improvement, he thought the fatality which had overtaken Nurse Preston should make for better treatment, and that the nurses should have the best possible conditions when dealing with contagious diseases." Other members of the Board endorsed the testimony of Mr. Walker as to the confidence and esteem which were entertained for Miss Preston, and, finally, Mr. James M'Donnell said he hoped that "if" there had been any insufficiency in the treatment of the nurses in the past, the matter would have more of their attention in the future. This is all very well, and we are glad to learn that five more nurses have been engaged to cope with the typhoid epidemic in the hospital; that temporary quarters of a sanitary character have been provided for nurses sleeping in the hospital, in lieu of "the culture bed of typhoid;" and that each of the nurses convalescent from typhoid is to be granted a month's leave of absence. But it is none the less deplorable that it should have required the death of a nurse to arouse the Belfast Guardians to a sense of their duty both to the patients and to the nursing staff under their care.

### NURSES' HOME AT FROME.

The Nurses' Home attached to the Victoria Hospital at Frome, which has just been opened and given the Diamond Jubilee Memorial of Queen Victoria, is a commodious and admirably arranged portion of the building. Situated on the north side of the hospital, it contains ample sitting and bedroom accommodation for the nursing staff, and has been suitably furnished. Not the least satisfactory feature in connection with the opening was that the nurses themselves collected no less than £125. This substantial result may be commended to the attention of the managers of other charities, and thanks to the response thus given the cost of the entire building has not only been defrayed, but there is a balance of upwards of £50 in hand.

### THE SCARCITY OF NURSES AT THE WORKHOUSE INFIRMARIES.

There is clearly an increasing disinclination on the part of nurses to accept employment under the Poor Law Guardians. The post of superintendent nurse at Bishop Stortford Workhouse Infirmary was advertised as vacant the other day and three candidates were selected. But as soon as they were informed of the fact they all wrote expressing their inability to attend before the Guardians. This would appear to be an additional argument in favour of the plan adopted at Gainsborough of engaging nurses upon the strength of their testimonials and not requiring an interview. At Bath the Guardians have been driven to the expedient of promoting a probationer who has only been in training eight months to the position of assistant nurse because they did not receive a single reply to their advertisement. At another workhouse infirmary in Kent an advertisement for a head nurse produced only one application, and the applicant declined to appear before the infirmary committee; while in Devonshire so many Boards of Guardians find it impossible to obtain, or retain, the services of nurses that a conference on the subject has been suggested. At a meeting of one of the Devonshire Boards a report was read of surplus rations supplied to a nurse having been thrown out of a back window into a field. The Guardians resolved to "let the matter slide," because "the chances were that if they said anything to the nurse on the subject she would resign, and there would be a vacancy." Of course, it is very wrong for a nurse to throw either surplus or other rations out of the window, unless they are clearly not fit for consumption, in which case the fire, or the dust-bin, is the proper place; and the idea of nurses being able to hold Guardians in terror because of the inadequacy of the supply is not less objectionable than that of Guardians failing to treat nurses with proper consideration. However, the situation is evidently becoming so acute that the Local Government Board will be compelled to intervene, in which event the changes in the position and treatment of nurses in all Poor Law work, on which Miss Twining insists in another column, may at last be seriously considered.

### NURSES AT ALNWICK CASTLE.

The Duke and Duchess of Northumberland, in accordance with their annual custom, have been entertaining the nurses of the County Nursing Association at Alnwick Castle. Each nurse was allowed to take a friend with her. There were about 100 guests, about 40 of whom were nurses, including seven Queen's Nurses. The party arrived at the Castle at 11 A.M., the Duchess herself receiving them, and were shown through the beautiful rooms. They then partook of lunch, and afterwards attended a delightful theatrical performance. Subsequently they went over the gardens and grounds. Tea was served at 4 P.M., and they left Alnwick on their homeward journey by the 5 o'clock train very much pleased with their day's outing. The Duchess remained with her guests the whole of the day.

### NURSING AT PAU.

With reference to an inquiry in our columns last week, which was evidently prompted by the idea of starting a nursing home at Pau, a correspondent says that there is "A Dublin Nursing Home" in the town, and that an English nurse, who is a masseuse, resides at Pau. She adds significantly. "There is very little work for English nurses there, except for about two or three months in the spring."

### WHY FARMERS' DAUGHTERS?

At a recent meeting of the Joint Committee of Management of the Westmeath County Infirmary the warden of Wilson's Hospital said that "it would be an admirable thing if the daughters of farmers could be afforded an opportunity of becoming certified trained nurses in local institutions, so as to avoid the expense of qualifying themselves in Dublin for the very lucrative and advantageous positions open to the profession." Ultimately, a resolution in favour of certificates being issued to nurses trained in Irish county infirmaries and workhouse hospitals, so that they may be eligible for appointments under Poor Law Boards, was carried. On this point there is nothing more to be said until the question of inspection by the representatives of the Irish Local Government Board has been determined. The marked reference to "the daughters of farmers" will strike some English readers as peculiar, but they were probably singled out by the warden of Wilson's Hospital because they are a large class in the sister island for whom an opening is badly needed, and also because they are, generally speaking, strong and capable. It is, however, most essential that they should receive proper training, and not be allowed, as has sometimes been the case in the past, to "nurse" patients in workhouse infirmaries before they have acquired even an elementary knowledge of nursing.

### NURSING ASSOCIATIONS AND ADVERTISEMENTS.

A correspondent, who encloses an advertisement cut from a local paper, according to which visiting nurses "will undertake dressings, douchings, enemas, injections," etc., at so much a visit, asks if nothing can be done to prevent "such indecent advertisements appearing." She adds: "Surely if the public want a trained nurse it is taken for granted that she knows her work without having to go into details in advertisement." There are certainly some details which it is quite unnecessary to include in an advertisement; but though the public would not doubt that a trained nurse is capable of assisting at a serious operation, they might hesitate before requesting her to attend them for duties which are of everyday occurrence. For this reason we are scarcely inclined to blame a nursing institution for signifying a little specifically the kind of service the nurses are willing to perform. Still it is not always necessary, even in an advertisement, to call a spade a spade.

### ASYLUM WORKERS.

The examination, under the auspices of the Medico-Psychological Association, for the certificate in nursing and attending on the insane, will be held on Monday, November 4th, and the last day upon which candidates can enter their names is Monday, October 6th. It may be worth while to repeat that the possession of this certificate gives a claim to increased remuneration in at any rate a large number of public asylums. In his address at the meeting of the British Association this year Dr. J. B. Spence, referring to asylum administration and nursing, said: "No amount of ability on the part of the leaders will suffice if the rank and file do not know how to carry out their instructions in an intelligent and efficient manner. In order that this may be the case, systematic training is absolutely essential, and while one is grateful for the progress already made, and for the large number of reliable and experienced men and women we have now on the nursing staffs of our asylums, there is still much to be done before finality in this direction will have been arrived at." This is very true, and the certificate granted by the Medico-Psychological Association is but an initial step in the right direction.

### LITERATURE FOR INVALID SOLDIERS.

A correspondent writes: "A friend of mine has a large number of magazines and other books which she is anxious to send out to the soldiers in South Africa. Her idea is to get some one to take them who is going out, and who, at the other end, could give them to people who would send them to a transport ship coming back to England with wounded. It seems that these convalescing men have nothing to read, and find the voyage very dull. Do you think that any nurse, or nurses, would be willing to take some of the books and see that they get into the right hands at the Cape?" If any of our readers are going, or returning, to South Africa, and care to undertake this little labour of love, we shall be glad to hear from them, and put them into communication with our correspondent.

### Lectures to Nurses on Medicine and Medical Nursing.

By NORMAN DALTON, M.D. London, F.R.C.P., Physician to King's College Hospital.

LECTURE XX.—BRIGHT'S DISEASE (*continued*).

IN the last lecture I said that, apart from the urine, the symptoms of Bright's disease could be divided into two groups, namely, those caused by the loss of albumen, and those due to a poison in the blood. Now the varieties of Bright's disease in which the urine contains most albumen, are "acute nephritis," the "large white kidney," and the last stage of the "lardaceous kidney," so that it is in these cases that we find most dropsy and most weakness. Nurses should, therefore, be constantly on the look out for dropsy, and its appearance in any case is a certain sign that the condition of the kidney is worse. For instance, acute nephritis is rather a common complication of scarlet fever, and sets in from three to six weeks after the beginning of the illness. In many cases, if the urine be examined every day, small traces of albumen may be found, indicating that the kidney is affected but not severely. Now, in such a case the occurrence of dropsy would indicate that the nephritis had become severe, and it would be found that the quantity of albumen had greatly increased. In fact, in cases of scarlet fever in which the urine is not frequently examined, the occurrence of dropsy may be the first sign to attract attention to the kidneys. Nurses must remember that renal dropsy occurs particularly in the eyelids, the genitals, the lumbar region, and the legs. Also in the above-mentioned cases, nurses must bear in mind that the patient is extremely weak. They must note any faintness or shortness of breath, and must carefully guard the patient from any exertion.

The symptoms of uræmia comprise headache, cramps, vomiting, dyspnœa, hæmorrhages, convulsions, and coma. The varieties of Bright's disease in which uræmic symptoms are most commonly met with are "acute nephritis" and the "granular kidney." In nursing renal cases, therefore, note should be taken of any of these symptoms. As regards the headache, the position of the pain and its duration are important. As regards the vomiting, any feeling of nausea should be inquired for, and the kind of food last taken should be noted; and, as regards the dyspnœa, it is important to observe whether cough be also present, and if the cough be attended by expectoration; for the lungs may become œdematous even when there is no general dropsy, in which case the cough will no longer be dry. In all cases of uræmia the closest watch should be kept for anything like a convulsion, and the slightest twitch of the facial muscles or fingers be most carefully reported. Again, drowsiness is important, as it is so frequently the beginning of a fatal coma.

It will have been observed that in "acute nephritis" we get both the symptoms due to loss of albumen and those due to uræmia. This is due to the fact that all parts of the kidney are, in bad cases, suddenly and severely affected, so that there is a great leakage of albumen and a great diminution in the performance of the functions of the kidney. In addition the kidney is unable to excrete even water, so that the quantity of urine is greatly diminished, which possibly increases the tendency to dropsy. Lastly, in cases of acute nephritis due to scarlet fever and cold, there is often blood in the urine. Hence the typical urine in these cases is very scanty in amount, highly charged with albumen and "smoky" from the presence of blood; while dropsy and weakness, and headache, with all the uræmic symptoms, are apt to be present. On the other hand, in the "granular kidney," the uræmic symptoms are usually present in great severity, without any dropsy. This is partly due to the fact that the albumen does not leak out of the blood vessels into the renal tubules, so that the urine contains little, and sometimes no, albumen. Also, the urine is very abundant, which of itself tends to prevent the occurrence of dropsy. So that in the case of the "granular kidney" the urine is abundant, pale, and of low specific gravity, and contains but little albumen, while uræmic symptoms are very prominent. The patients have a dusky, rather dried up appearance, and an anxious expression, and they may be extremely weak, although they are losing no albumen to speak of.

It is interesting to know that one form of Bright's disease may pass into another. If a patient contracts acute nephritis and does not get better, the affection becomes chronic. Usually the kidneys become "large white kidneys," and there is much loss of albumen and much dropsy. If the patient survives this condition for many months the kidneys become "granular," the dropsy clears up, and the loss of albumen is little, but uræmic symptoms set in. Lastly, the "granular kidney" may at any time become acutely inflamed, in which case there is much albumen (and sometimes blood) passed and the dropsy recurs. In all the varieties of Bright's disease the arterial tension becomes high (unless the debility be extreme), so that the arteries feel overfull; and, in old cases, they may feel like whipcord from thickening of their walls. The heart, especially the left ventricle, becomes very big, and beats very forcibly; but this extra power of the heart is apt to fail rather suddenly from degeneration of the heart's muscle. Lastly, there is a peculiar form of inflammation of the retina which occurs in Bright's disease. It is called "albuminuric retinitis," and causes great deficiency in vision. It is well known that patients have consulted an oculist for defective vision and he has been able to tell them that they have Bright's disease by merely examining their retina with the ophthalmoscope.

The modes of death in Bright's disease are also interesting. With the "large white kidney" death is due mostly to the loss of albumen, *i.e.*, the heart may gradually stop from sheer weakness, or dropsy of the lungs may cause suffocation or inflammation of the dropsical lungs, or of the œdematous larynx, or erysipelas of the dropsical legs may determine the fatal issue. On the other hand with the "granular kidney" death usually occurs from the poisoning, *i.e.*, from uræmic convulsions or coma. But another frequent cause of death with a granular kidney is the rupture of a blood vessel in the brain. This is brought about by the degenerated state of the arteries, and all patients with this affection should therefore be warned against sudden violent efforts or outbursts of passion. It will follow from what has been said that in acute nephritis death may be brought about either by the loss of albumen (dropsy, etc.) or by uræmia. In all cases of Bright's disease pericarditis is liable to occur, and it is very fatal.

Bright's disease is a very fatal malady, but such extraordinary fluctuations occur in the patient's condition during its course that it is never right to say at any time that the case is hopeless. For instance, in acute nephritis, death may appear imminent from coma or convulsions, and yet, under treatment, these symptoms may pass off and the kidneys may get perfectly well. In the chronic cases the kidneys never get quite normal again; but even in the "granular kidney" the gravest uræmic symptoms may come on and pass off again, leaving the patient in fairly good health for months. Also in the "large white kidney" the patient may appear to be in the last stages of exhaustion from dropsy when suddenly the albumen in the urine may diminish and the dropsy may clear up. If the case be a fairly recent one and no uræmic symptoms appear, this diminution of the albumen may be considered an indication of a true recovery; but as a rule, it merely means that the kidney is becoming granular (as has been mentioned above), and, after some months, during which the patient gains strength and feels immensely better, the urine becomes very abundant and headache and other uræmic symptoms set in.

### War Nurses at Southampton.

By a Correspondent.

Southampton is a most confusing place to a stranger, and the oddest thing about it is the calm and apparently irresponsible manner in which the passenger trains cross the public road every now and then. It is true a bell is rung loudly, but until I saw the train slowly curving out of the station and into the docks, I did not know what the bell meant. Of course, all traffic was stopped for the time being.

The spot for which I was bound was "Shed 25," for this is where the embarkation duties are performed, and where all the home-coming nurses from the war have to report themselves before entraining for London or elsewhere. Here they leave their names, and the addresses which will find them during leave of absence; in many cases, especially if the nurse is a colonial, a mere postal address is given, perhaps at a bank or some house of business where she may have a relative or friend. Not far from this shed an orderly met me, and conducted me to the medical officer in charge of the embarkation duties. "This is the office," he said, "where all the clerical work is done, and from which those familiar blue papers are sent out."

It was a minute place, with just room for two or three chairs and a writing-table; next to it was another office where a couple of orderlies were at work.

"What is the first thing the nurses do," I asked, "when the ship comes in?"

### Lady Dudley's Fund.

"They report themselves here," was the reply; "and my business is to tell them, first of all, about Lady Dudley's arrangements for those who are not going directly to friends. Then a wire is sent to the rooms in London, and the nurses go off by train. That is, those who are in health; and I must say that excellent health has been the characteristic of all who have recently arrived. But if they are ill I telephone up to Miss Mocatta's Nursing Home, in Southampton, and she sends down an ambulance in which the invalid is at once removed to Cumberland Place to be nursed. I see that Lady Dudley is appealing for funds to carry on her work; it is of immense value to the nurses, I can assure you."

### A Nursing Department in the R.A.M.C.

"I wish," the Colonel continued, "that 'The Hospital' would lay great stress on the need for a nursing department in the R.A.M.C. That is a scheme which I am most anxious to see carried out, and I should like to hear what army nursing sisters think about it. Perhaps they would like to sweep the orderly off the face of the earth, and run the military hospitals themselves? At any rate, if we are to have the present system—which has its faults—a nursing department seems to me of the greatest necessity. How can anyone expect a loafer who has been hanging round the public-house until the recruiting sergeant picks him up, to turn into a good nurse all at once? It is impossible. What we want is a plan by which a man will not only receive thorough and efficient ward training, but will be able to rise in proportion to his abilities. Some people suggest turning a detachment of orderlies into the wards of the London hospitals; I wonder how the sisters and nurses would like it? This war has brought out the weak points in the present system; and the lack of adequate training for orderlies is one of the most striking."

"In fact, if we must have orderlies, let them be the best that can be had?"

"Exactly."

### Sisters' Quarters on the "Avoca."

Whatever may be the faults of the system of Army nursing, there was nothing to complain of in the arrangements for the nurses' comfort in the hospital ship which I had come to see. This was the *Avoca*, No. 6 Hospital Ship, which was then in dock, though due to sail on the 20th. I was guided across the intricate ways of the dock-yard by a sergeant, on whom, as on a stormy petrel, I fixed one eye, while with the other I kept a sharp look-out for trains. As I stood on the bridge and watched the great white hull, with a huge red cross painted on the side, being towed along by a steam tug, it seemed as if a whole street were going slowly past, with sailors, both English and Coolies, shouting from what might have been the roofs, so far were they above where we stood. There was a good deal of throwing of ropes and rushing backwards and forwards, and finally a gangway was placed aft, and we climbed on board. Here an orderly in khaki with a bunch of keys met us, and conducted us to the hospital part of the vessel. It was still in the hands of the workmen, for the entire ship is scraped and painted each time she comes into dock; and though to my unpractised eyes all seemed in hopeless confusion (for repairs to her propeller were also being carried out), we were told that the work was nearly completed. In the midst of this carpentering and painting a group of officers was lunching in the saloon, while dusky Lascars in white turbans ran to and fro with dishes, one of whom, in answer to the Colonel's question in Hindustani, said he was from Calcutta and a Mussulman.

"The Sister Superintendent has a better cabin than the P.M.O., I think," remarked the Colonel, as the door was unlocked and we peeped inside. It was very tiny, but quite large for a cabin, I was assured; the bunk was on one side, and there was a chest of drawers and an easy chair, as well as room to turn round comfortably; also a bath-room close by. The nurses' cabins were on the same plan, in a double row leading off from the sister's room; the only difference was that they looked a trifle smaller, and that in one or two we noticed an upper berth. They were all painted white, and looked very clean and inviting. We did not look into all, but judging from what I saw everything seemed to have been arranged with a view to as much comfort as possible in a limited space.

### In the Wards.

Next we went into the "wards," and here my guide told me he was very anxious to find out which were really most suitable for the purpose, swinging cots or fixed ones.

"The advantage of a swinging cot is that you can have it fixed if you like," he said, "whereas you cannot make a fixed one swing." I wondered which the sisters preferred. The sides of these swinging cots take down like those of a child's crib, and there is a little tray fixed on for holding bottles or glasses, etc.

"All the hospital ships now are having single tiers of cots," the Colonel continued. "It has taken about two years to get this carried out, but at last it is an accomplished fact. Here," opening a door, "are some of the old-fashioned sort, they are used now by orderlies, and they do very well for healthy people but not for sick ones."

These were an upper and a lower berth fixed together, something like the beds provided in the Salvation Army shelters. The difficulties of nursing in either must have been supreme, for in attending to the patient in the lower berth the nurse's head could hardly help knocking against the upper one, while as to the upper berth, I had only to recall what one of the nurses told me some months ago, about sponging an enteric case at arm's length, stumbling backwards off the box she was standing on, whenever the ship gave a lurch. Nurses coming home on hospital ships wil welcome the news that these upper berths are being done away with.

We next visited the operating theatre, and I was shown the electric fans which are such a comfort in the tropics, and the warming apparatus, so necessary when the climate changes and the heat is left behind, and last but not least, the electric kettle, which is such a convenience at all times.

The Stretcher Corps.

As we left the ship, passing a group of Lascars, squatting round their tin cans and chattering like monkeys, I remarked that several nurses had made special mention of the clever way in which stretcher-cases were removed from the vessels.

"That," said the Colonel, "is done by a special corps of orderlies, and great pains have been taken to make the work of removing the sick as painless as possible."

Nursing the Nurses.

Later in the day I had a pleasant chat with Miss Mocatta, whose nursing institution is in one of the pleasantest parts of Southampton; she told me that she always liked to give the invalided nurses a room to themselves, with a bright and cheerful outlook, for as a rule they came to her thoroughly broken down in both body and mind from the strain of work, and quiet and rest were very essential. She had only had a small number from South Africa to nurse, but her predecessor at Norfolk House had a great many during the rush of invalids from the front.

Finally, after a kaleidoscopic view from the top of an electric tram, of the town so full of associations to our war nurses, I left Southampton *en route* for London.

### Life in a Navvy Hospital.

By a Nurse.

It fell to my lot to work among the navvies for eight months. The last two years or more nursing at the front, and the Chinese and plague troubles, have so occupied everyone's attention, that we are apt to overlook that other large army of hardworking men, brave men, too, and as heroic as any who face death on the battlefield. Navvies have the name of being rough, rude, brutal—almost everything that is bad—but in eight months' personal contact with them I found much that was good, brave, noble and unselfish.

My Introduction to the Navvies.

In fear and trembling I received my summons to one of the three temporary hospitals that had been erected on the "line of works." I reached my destination at 6 p.m., and having gained the information that the hospital was "but a step, and yer couldna' miss un," I left my luggage to come on an engine "up the works," and walked on to find the hospital. After passing through the town I found myself in a lane carpeted with thick mud into which I sank up to the tops of my boots—and fairly stuck at last. Wondering how I should get out and what I should do in this uncomfortable dilemma, I saw a group of what I thought at first "dreadful-looking men" emerging from a small public-house, and heard a burst of amused laughter and a lovely brogue saying, "Ye'll be wanting to be puttin' on yer top-boots next time yer comes this way, nurse," and a knobbly stick and big earth-covered hand were stretched out to my help, and I was hauled out on one side of the lane where big stones, unseen before for the mud that covered them afforded firmer footing. That was my introduction to the navvies and it was a specimen of their behaviour on most occasions Good humoured chaff, but always a helping hand if necessary; no change of dress Sunday or week-days; always the public-house when "off duty," but also always a shilling to spare for a "mate" who by reason of an accident was out of work. I need not have been timid at going amongst them, and never was after that first sight of them, for on no occasion did one man out of the 7,000 ever behave to me disrespectfully or discourteously.

On Duty.

The lady superintendent received me kindly, and after tea I was shown my quarters, and asked when I could go on duty. I found I was to take sole charge at night of thirty-three patients, in three wards, and I, being young and strong, said I could go on that night. Consequently at 8.30 p.m. I arrived on the scene and went round to view my new patients. There was an out-patient department and a surgery, a neat little ward kitchen with the three glass-panelled ward doors leading out of it. The long ward was the centre one; it contained 25 beds, but on this my first night it held only 19 patients. The left-hand ward held five beds chiefly for cases that required night attention, or more warmth or quiet than could be obtained in the long ward; and the right-hand ward was smaller still and more private. It had three beds, and was kept chiefly for operation or very critical cases. It was called by the men the "guard-room," chiefly because one man who had been a soldier got transferred there one night when his very noisy D.T. fit "came on him."

"Lucy Jean."

The cases were for the most part accident cases, every kind of accident that could possibly happen to "careless man"—and the most careless and reckless is the English working man—being brought in to that tiny hospital from a crushed thumb, jammed in a chain while filling waggons to a broken spine; from a trifling sprain, or gash, or scald, to a poor wreck blown nearly to bits by careless "ramming" while blasting up the rock. One poor fellow was carried in charred all over, burnt nearly to death through "skylarking." Lying before the fire in his hut some of his mates had poured a can of a particularly inflammable oil which they called "Lucy Jean," down the little funnel chimney to "rouse him out," meaning only to cause the fire inside to flare up with a sudden light, but they caused his death. Heavy and tired with work, he had thrown himself before the fire on the floor and slept. Lucy Jean had roused him, indeed, but his clothes had caught fire, and he was brought in to me in a dreadful condition. The doctor and a policeman helped me to swing him in a hammock in a 6-foot-bath of water, for he could not touch the bed. He lived in that condition four days. We sent to Scotland for his wife, but he died an hour before she was able to see him.

An Accident from Rock-Blasting.

Another busy night, I had two operations and a terrible accident from rock-blasting to see to. This last we considered one of our masterpieces. A young fellow of 25 foolishly went to see why a charge had not exploded, and began "ramming" it, when it suddenly went off, blowing his cheeks clean open, and his teeth out. His fingers and arm were gashed about with splinters of stone, and the marvel was that he was not killed. Every bed was full, so, as he was getting "collapsed," we laid him on the rug in front of my ward kitchen fire, and "worked at him" hard. The way the doctor carefully replaced his shattered cheeks, stitched the "bits" of his lips, picked out the splinters and the teeth, was splendid. In less than four weeks most of the scars were healed. This patient lost the sight of both eyes, but when his beard grew he looked almost handsome again, for he had been quite a good-looking young fellow. He was allowed a pension for life, and to our great joy, the girl who had "walked out" with him, married him, and they were quite comfortable and very happy.

A CATASTROPHE TO A TRAIN.

My biggest casualty was when a train of small waggons upset as it was crossing a "gantry"—*i.e.*, a hasty sort of bridge, at least that is what I always understood it to mean. Ten men were injured and brought in, the ward was full of policemen, nightwatchmen, and the "mates" of the injured men, who were in their own way very helpful in taking off the heavy mud-clotted clothing and great wooden-soled boots of my new patients. One died, all the others recovered. It must have been something like nursing at the front I imagine. We had to make use of all kinds of things not usually known to the nursing world. Our "hot bottles," for instance, were bricks, heated in the oven and wrapped in brown paper; our splints were "all sorts," they were often padded while the patient "waited," and we didn't stop to sew the padding, but I think the splints were none the worse for that. It was easier to strip them and burn the old dressing. All we did was to form the padding of tow and absorbent or any other wool, and fasten two bands of strapping round and our splint was ready! The worst time we had was when an epidemic of influenza broke out. Many died from that and alcoholic pneumonia; otherwise, our patients for the most part made good recoveries, and I think that though, of course, it was due a great deal to the nursing, yet the cheery spirit and hardiness of the men must have had much to do with the quickness of their recoveries from such accidents as I have rarely seen elsewhere.

SPLENDID PRACTICE.

It was splendid practice for surgical work, and I have never regretted the short time I spent among these men, who seem to live a life of their own apart from town life, village life, or that of the ordinary working man. I learnt more of our great works—such as the Blackwall Tunnel, the Severn Tunnel, the building of the Forth and Tay Bridges, England's great waterworks, etc.—from these men than I ever did from books. Eight miles from where I am now staying there is one of these large navvy settlements. The men are engaged in making the reservoir for the Birmingham Waterworks; they have their own village built of huts, their own hospitals—which I mean to go over one day, hoping to recall memories of my own life in a navvy hospital—their own schools and mission hall, just as ours had, and alas! also from what I see of the men who occasionally find their way down this valley, their own craving for strong spirits and power of satisfying it. They earn good wages, but spend nearly all in drink.

### Appointments.

BETHNAL GREEN NEW INFIRMARY, CAMBRIDGE HEATH.—Miss Alice M. Garratt has been appointed superintendent nurse. She was trained at The Infirmary, East Dulwich, and for two years has held the post of sister at the same institution.

DENES FEVER HOSPITAL, SOUTH SHIELDS.—Miss Ada Louisa Davison has been appointed charge nurse. She was trained, for three years, at the Royal Infirmary, Newcastle-on-Tyne, and Denes Fever Hospital, South Shields for 16 months. Since then she has been charge nurse at the South-Eastern Hospital, New Cross.

EAST GRINSTEAD UNION INFIRMARY. — Miss Harriet Hillson has been appointed superintendent nurse. She was trained at Greenwich Union Infirmary.

FEVER HOSPITAL, YOKER, NEAR GLASGOW.—Miss Nellie Cotton and Miss Audrey Thurston have been appointed charge nurses. Miss Cotton was trained at the Isolation Hospital, Bagthorpe, Nottingham, and has since been assistant nurse at the Fever Hospital, Yoker, the Women's Hospital, Nottingham, and the Accident Hospital, Mansfield. Miss Thurston was trained at Winchmore Hill and the Brook Hospital, London, afterwards doing private nursing. She has also been nurse at the Evan Fraser Hospital, Sutton, and charge nurse at Willesden Isolation Hospital, Willesden.

GENERAL HOSPITAL, TUNBRIDGE WELLS.—Miss Edith Louisa Ramsay has been appointed night sister. She was trained at Guy's Hospital, London.

LAMBETH INFIRMARY.—Miss Emma Mary Byles has been appointed matron. She was trained at Addenbrooke's Hospital, Cambridge, and St. Thomas's Hospital, London. Afterwards she was engaged in private nursing at Cambridge for a year, was night superintendent at Addenbrooke's Hospital for a year, and ward sister for six years. From 1899 to the present time she has been assistant matron at St. Saviour's Infirmary, East Dulwich.

LEIGH-ON-SEA.—Miss Helen Browne has been appointed district nurse. She was trained at Queen Victoria's Jubilee Institute for Nurses, Worcester Hospital, and Cardiff, and she has since been district nurse at Coleford; Finedon, Wellingborough; and Colchester.

LLANDRINDOD WELLS HOSPITAL.—Miss G. Hopper has been appointed matron. She was trained at Addenbrooke's Hospital, Cambridge, and St. Mary's Hospital, London. She has since been charge nurse at the Royal Mineral Water Hospital, Bath; sister of the women's medical and ophthalmic wards, and sister of the men's surgical and accident ward at East Suffolk Hospital, Ipswich; and matron of the Cottage Hospital, Bushey, Herts.

MADELEY UNION INFIRMARY, SALOP.—Miss Charlotte Hodgetts has been appointed nurse. She was trained at the Borough Fever Hospital, Birmingham, and has since been nurse at the Lichfield Union Infirmary and the Tamworth Union Infirmary.

NEWPORT COUNTY HOSPITAL, MON.—Miss T. Hay has been appointed night superintendent; Miss J. Rippon and Miss M. Hendry sisters; and Miss Cummings and Miss Robinson staff nurses. Miss Hay was trained at St. Bartholomew's Hospital, London, and was on the staff for four years. Miss Rippon was trained at Wigan Infirmary for three years, and at Cardiff Sanatorium for two years, subsequently acting as theatre sister at the General Hospital, Sheffield. Miss Hendry was trained at Crumpsall Infirmary, Manchester, and has since been sister at Wigan Infirmary, private nurse at Nice, and charge nurse at Newport Hospital. Miss Cummings was trained at the Hahnemann Hospital, Liverpool, and has since been engaged in private nursing. Miss Robinson was trained for three years at the Royal Infirmary, Edinburgh.

ST. MARY'S HOSPITAL, MANCHESTER.—Miss Alice Hulme has been appointed sister. She was trained for three years at the General Infirmary, Chester, and was afterwards staff nurse; she has since done private nursing on her own account, has been charge nurse at the Carnarvonshire and Anglesey Infirmary, Bangor; assistant maternity nurse in connection with the District Nursing Association, Llanelly; and charge nurse at the Isolation Hospital, Chester.

SWANSEA GENERAL AND EYE HOSPITAL.—Miss Rae Boden has been appointed theatre sister. She was trained for three years at St. Mary's Hospital, Manchester, where she has since been sister for three years. She has also been head nurse, for 16 months, at Bromley Cottage Hospital.

SWANSEA UNION INFIRMARY.—Miss Martha Hill has been appointed charge nurse, and Miss Mary Ann Taylor has been appointed certificated nurse for day and night duty. They were both trained at Bristol Workhouse Infirmary. Miss Hill has since been superintendent nurse at Keynsham and Tredegar Workhouses, and Miss Taylor has since been nurse at Wakefield Workhouse Infirmary and in private nursing.

VICTORIA HOSPITAL, DEAL.—Miss Edith James has been appointed staff nurse. She was trained at the Dreadnought Hospital, Greenwich.

WEST CORNWALL MINERS' HOSPITAL, REDRUTH.—Miss Margaret Brewster has been appointed matron. She was trained at the Royal Southern Hospital, Liverpool, where she was afterwards staff nurse. She has since been sister in charge at the Royal Eastern Infirmary, Wigan, and matron of the Borough Hospital, Ashton-under-Lyne.

### The Nursing of Tropical Dysentery.

By a Sister in a Colonial Hospital.

(*Concluded from page 331.*)

### Close Care Essential.

The nursing of a dysenteric patient demands almost as much forethought and attention as one of enteric fever, and close care should be exercised throughout the case. The patient should be kept in the recumbent position until the doctor definitely allows him to sit up and move about, and, especially in cases where much blood is passed, the bed pan should take the place of the commode, lest the act of sitting up on the latter contrivance induce fatal syncope—as I have seen. Ice may be given to allay the excessive thirst and dryness of the mouth which are usual accompaniments of the disease. Most medical men permit also orange juice, and some of this is generally at hand in tropical or sub-tropical countries. All nourishment should be fluid, and be given in small quantities at frequent and regular intervals, subject to the exigencies of the ipecacuanha treatment while that is being followed. Hot opium stupes are sometimes called for for the relief of abdominal pain, and when there is flatulent distension or tenesmus turpentine, fomentations afford an effectual palliative in almost all cases. A flannel belt or roller may also be worn with advantage, if it does not cause discomfort to the patient. The nurse should sponge the body and limbs with hot water and some bland soap every day, taking all the customary precautions to avoid unnecessary exposure, and thus prevent chill, especially of the trunk. Some disinfectant should be put into the bedpan before use each time, and also some deodorant, perhaps the best being eucalyptus oil for the latter purpose, for all dysenteric stools are very pungent and disagreeable. A little coffee seed charred in a hot shovel in the room is also an excellent countervailing resource in this respect. Occasionally the doctor may order a motion to be preserved for examination with the microscope; in such event, of course, it must not be treated with anything, but be cautiously poured from the bedpan into a urine glass or other convenient vessel with a well-fitting cover, and be temporarily deposited in a safe place out of doors.

### Infants.

In the case of infants, dysentery is sometimes more difficult to deal with than in the adult, especially when occurring about the period beginning of the dentition period. The child's diet is of the greatest importance, and must be administered in very small quantities every hour or two hours. Vomiting is a serious complication and is easily induced by an overdose of milk, which comes up coagulated in hard curdy masses. For this reason limewater is often added, in quantity according to the age of the child, and not infrequently it becomes indispensable that milk should be eliminated from the dietary altogether; in which event chicken jelly, bread jelly, sherry whey, albumen water, and even small quantities of beef tea or mutton broth must be substituted. The medicinal treatment in such cases is usually begun by a teaspoonful of castor oil, or by successive small doses of a castor oil emulsion made with gum arabic, sugar, and dill water, and followed by a bismuth and salol emulsion, containing a fraction of a minim of laudanum or not, at the discretion of the doctor, but of no other person under any circumstances. Or very small doses may be prescribed, with or without bismuth or salol as the case may be. As in enteric cases, patients convalescent from dysentery will want to eat all sorts of abominations that are not fit for them, long before the doctor will dream of permitting such indulgences. It is most important that time be given for any ulcers remaining in the intestinal wall to cicatrise thoroughly before solid food, especially meat, be taken.

### Melanesian Patients.

It is remarkable to see what Indians and Fijians will pull through; on the other hand Melanesians often succumb very rapidly. They make no effort to rouse themselves, but apathetically await events. With these people it is not an uncommon thing to find that your patient has left his bed, and when you look for him to discover that he has crawled beneath the ward which is built of wood, on piles three feet high, or is lying under an adjoining bush, and when remonstrated with, he says, "Oh, I am going to die; it does not matter." Fijians, too, are inclined to be fatalists, though you can sometimes persuade them to submit to treatment, and when they can see any good result they are satisfied. But unless there is something tangible for them to see or feel within 24 hours of the commencement of the treatment, it is very difficult to make them believe that any thing you can do is of benefit to them; and they then ask to be allowed to die in their own villages, so that they may enter purgatory or elysium by the right channel. Of course, an acute case is obliged to stay in hospital, but others are allowed to go home. It is certainly better so for the hospital statistics, but one often wishes they would stay and have proper care until the end, for I am sorry to write that, through savage ignorance, the dying are often very much neglected by their fellow natives. Every argument is used to induce them to stay, but the wish of the patient and relatives is too often so obstinately pressed that it has to be deferred to.

### The Case of a Prison Warder.

I should like to quote two rather unusual cases. One was a Fijian who was admitted with an acute attack of the disease, with a temperature of 103°. For several days his temperature remained high, and in about two weeks, when the acute symptoms had subsided and the temperature had become subnormal, he had sudden severe pain promptly followed by loss of motor and sensory power in the right foot and leg, as high as the level of Poupart's ligaments. Pulsation could not be felt in the femoral artery, and the circulation ceased in the limb. Gradually the foot became gangrenous, and, as the man objected to operative treatment, he got rapidly into a septic condition. He expressed a wish to go home, a distance which would take four or five days by boat, to die there. Permission was refused as he was a prison warder and under definite discipline, but it was not before the line of demarcation was well above the knee that he consented to have the limb amputated. This had to be done at the hip joint. It was certainly almost a hopeless case. The patient was very collapsed after the operation, and his previous condition was so bad that we scarcely expected him to rally; but he improved wonderfully, and is now in excellent health and perfectly happy and pleased with himself. When he is asked now what would have happened if he had gone home, his only reply is a smile.

### Presentations.

Isolation Hospital, Chester.—Miss Alice Hulme has been presented with a brass inkstand and photo frame from the nurses with many expressions of good wishes for success in her future work.

### Wants and Workers.

Will anyone having an invalid's chair to dispose of kindly confer a great boon on a poor woman who is unable to walk by giving or selling it cheap to her? Address Nurse Winterflood, 30 Retreat Place, Hackney, N.E.

### Echoes from the Outside World.

### AN OPEN LETTER TO A HOSPITAL NURSE.

THE Czar's visit to France is past and over, and, to the great relief of everyone concerned, it has been accomplished without an attempt from any wretched Anarchist to follow Czolgosz's example. Paris, of course, was sadly disappointed that the Czar did not honour the capital with a visit, but France generally seems very pleased that he should have reviewed the French Army as well as the French Navy and commended both. The Royal couple arrived on Wednesday after a very stormy passage, which somewhat incapacitated the travellers. The French warships were grouped around, and the visitors were met and welcomed by M. Loubet at Dunkirk, lunching at the Chamber of Commerce, and then proceeding to Compiégne. The Czarina was presented with a nosegay of heather gathered in the neighbouring forest, which must have been a pleasant change from the usual bouquet of exotics she is so frequently given, and then their Majesties drove to the Castle, the route being guarded by 8,000 troops. On Thursday the military manœuvres took place in the neighbourhood of Reims, and at the luncheon the Czar spoke in flattering terms of the French Army. The next day the guests enjoyed a quiet morning, followed by a christening, when the Czar himself proposed to stand sponsor to his Ambassador's infant grandchild. The grand review of the French Army was held at Betheny on Saturday. His Majesty rode on horseback attended by a picturesque Arab escort, but dismounted to witness the march past, which occupied some hours. At the close there were shouts from the populace of "Vive l'Armée!" "Vive le Czar!" "Vive la Czarinè!" "Vive la France!" etc. Later in the day the visitors left France for Kiel on their homeward journey.

ASSUREDLY the last week was a week of ill-luck to England. At home, comparatively close to shore and to those they loved, sixty-seven men were, well-nigh without warning, hurried into eternity owing to the disaster which overtook H.M.S. *Cobra.* The vessel was a turbine torpedo-boat destroyer on what may be called her first voyage. She had been made at Elswick, where she was seen and purchased by the Government, and on her way to Portsmouth to be enrolled in the Navy she encountered tempestuous weather off the Lincolnshire coast, is believed to have struck on a rock near the Dowsing Sands, and in five minutes had broken in half. So many men crowded into the whaler when she was launched that she upset, and all on board were drowned. But a few men succeeded in climbing into the dinghy and getting clear of the wreck. But the sea was still dangerously rough, and they had to proceed very warily, more especially as the boat already held nine individuals when it was only constructed to carry eight, and the rowers were surrounded with drowning men, who would naturally have clutched at their last chance of being saved. As it was three men—one of whom had injured his leg—clung on to the sides of the frail little boat, and it was not till more than an hour after the accident that it was deemed safe to pull them over the sides. Even then they had much to suffer before they were seen by a passing steamer—ten hours later—for the men were nearly all half-clad, and they were without either food or water on board. The chief engineer jumped from the *Cobra* as she sank and swam out to the dinghy, but Lieutenant Bosworth-Smith, an officer of great promise, who was in command, went down standing on the bridge with his arms folded. The *Cobra*, it is said, cost £70,000 to build, and her twin sister, the *Viper*, was lost off Alderney only a short time back. These destroyers are constructed so that they may be as light as possible, the steel of which they are formed is less than a quarter of an inch thick, and they go at a tremendous pace, so that they are only fit to be used in calm weather and very easily break. Moreover, it is said that Navy ships named after reptiles or things that sting are always unlucky. Be that as it may, it is strange that four *Vipers*—all wrecked in home waters—four *Serpents*, three *Lizards*, two *Snakes*, two *Wasps*, one *Adder*, one *Alligator*, one *Gadfly*, one *Crocodile*, one *Hornet*, one *Rattlesnake*, and one *Basilisk* have all gone to the bottom, to which melancholy list the *Cobra* is now added. A Mansion House Relief Fund for the sufferers has been talked of, but it is generally expected that the Admiralty and the shipbuilders will do all that is requisite.

IN South Africa the Boers apparently have only been waiting for the date—September 15th—fixed by Lord Kitchener for surrender to expire before breaking forth into fresh activity, unfortunately with rather grievous results to us. First Major Gough with three guns and about 180 mounted infantry were operating between Utrecht and Dundee, when they sighted about 200 Boers near the Blood River. Believing that they were unsupported—I wonder where the scouts were?—he pressed forward and found himself surrounded by the main body of Botha's force, about 700 strong. About twenty men, one officer, and the Colonel got away under cover of the darkness, but the rest of the little force, who were not killed, fell into the hands of the enemy, together with the three guns, which, however, had been damaged so as to render them useless as soon as it was seen how badly matters were going. General French has also had reverses to report. Smuts' commando, in order to break through the cordon of columns hemming him in, rushed a squadron of the 17th Lancers at Eland's River Poort, killing three officers and twenty men, wounding one officer and thirty men, and securing two guns. The enemy were close up to the Lancers, who fought most gallantly, before it was known who they were because they had treacherously dressed themselves in British uniforms, so that they might be mistaken for our own men. Lord Kitchener has sent a proclamation to both Commandant Botha and Mr. Steyn, in which he draws their attention to the fact that he has consistently endeavoured to bring the war to an end with due consideration for the burghers. But as now England is maintaining in idleness 35,000 men who are awaiting the cessation of hostilities, as well as 74,000 Boer women and children, Lord Kitchener appeals to the better nature of Commandant Botha to save his people from becoming in a worse condition, both morally and materially, by delaying the inevitable termination. He also reminds Mr. Steyn, to whom he recapitulates the same things, that though "his Honour" states that he places his trust in God Lord Kitchener cannot help thinking that as he first invaded British territory he must have overlooked the passage in God's Word which says, "They that take the sword shall perish with the sword." The only reply to these proclamations is the threatened invasion again of Natal by the rebels, which, though not an important movement, has necessitated the calling out of the Durban Volunteers.

YEARS ago, when an Eastern potentate was visiting this country, the question of the hour was "Have you seen the Shah?" The question of to-day is of a much more prosaic character, but none the less it greets one on every side, "Have you been vaccinated?" Occasionally it is not necessary to ask, because when the arm has arrived at a painful stage a careful observer can easily tell by watching a crowd the woman who is afraid of a rough blow. The recent victim of vaccination has a trick of turning herself sideways so as to put her sound shoulder to the wheel and protect the other, quite different from the square shouldered method of advance which is natural to most of us with two sound limbs. School girls and school boys have been "done" in large batches in many of the suburbs around London, and have enjoyed the process, because a very bad arm is a legitimate excuse for a holiday, if you are a day scholar, and yet you are not ill enough to be unable to enjoy the recreation. But those whose work needs two hands have naturally chafed a good deal at having to get along with one, though congratulating themselves that it is a "good thing over," and strongly counselling the next person they come across to go and do the same.

### Everybody's Opinion.

[Correspondence on all subjects is invited, but we cannot in any way be responsible for the opinions expressed by our correspondents. No communication can be entertained if the name and address of the correspondent is not given, as a guarantee of good faith but not necessarily for publication, or unless one side of the paper only is written on.]

### THE OFFICIAL ANNOUNCEMENT OF THE RESIGNATIONS AT CROYDON INFIRMARY.

"Certificated Nurse" writes: I would like to make it known that no error was made in my last letter to you, stating that 11 nurses had sent in their resignations "within the past two months." Since then another nurse has resigned, viz., Probationer-nurse Marsh, who sent in her resignation September 9, and who was not included in my calculation. This brings the number of resignations so far up to 12. Nurse Gordon, who left the infirmary along with Nurse Grey—as the result of the action of the visitors—on August 20, and who had not previously resigned, making a total of 13 nurses who have left the infirmary since July 24. The following is a list of the nurses who have resigned, with dates of resignations, and in some cases dates of leaving also:—Probationer-nurse Hunt, July 23, forfeited a month's salary and left the following day; Ethel Willoughby, July 1; Agitha Morris, July 23; Jenny Grey, August 14, left August 20; E. M. Gooch, August 14; F. Shird, August 14; Kathleen Pomeroy, September 6; H. Mathieson, September 8; May Willoughby, September 8; Marie Feetham, September 8; Annie E. Marsh, September 9. Both these latter nurses forfeited part salary and left September 14; and Nurse Curd (charge nurse of maternity wards), September 5, also forfeited part salary and left September 18. Miss J. Grey, who left the infirmary August 20, was not stated in the official report as having resigned. Again, the official report says: "The Committee recommend that Miss Gordon be paid her salary and a sum equal to the value of her emoluments, up to the 27th inst.," but they do not state that Miss Gordon refused to accept their offer; and that the board ultimately decided—for reasons best known to themselves—that Miss Gordon be paid her salary up to October 17th, and also the value of her emoluments from August 20th to the same date, which said offer was accepted. One of the guardians asks the chairman to explain why they have lost so many nurses. The reason is obvious to the whole of the nursing world. What a pity it is that men who hold this public office cannot, or will not, see that the whole truth, and not only part of it, is given in their official reports. Is it that they, as well as the public, are being deceived; or is it that the Croydon Guardians think "A little truth, however small, is better than no truth at all?"

### TRAINING AND NURSING IN POOR LAW INFIRMARIES.

"Miss Louisa Twining" writes: Those of us who have been considering this subject during the last 40 or 50 years, might be supposed to be satisfied with the vast improvements that have been made since the time when no arrangements were attempted for the care of the sick, and no help was provided beyond what could be given by their fellow-paupers. But we are not satisfied, and we still ask for more to be done, in ways which we consider practicable. Our Workhouse Infirmary Nursing Association which was the first to organise and carry out a system of trained nurses for Poor Law work, may claim to have acted as pioneers in the much-needed reform, but the committee never could have aspired to do all that was wanted, or to do more than make a beginning. The Association has been recently somewhat blamed for stopping this work, and ceasing their endeavours to train nurses for the Poor Law, but surely such criticism is unreasonable. How was it possible for a small voluntary society to undertake the work of a State Department which extended throughout the whole country, with the limited resources of at most a few hundreds a year, while 600 State institutions might be claiming our help, and many, indeed, had long been doing so, far beyond our means of assisting them. When the law was passed four years ago it became evident to us all that the task must be given up, and the duty thrown upon the department which had issued the rule, and surely may be considered ultimately responsible for carrying it out. So much for the past action, but what about the future which is now before us, and which demands our earnest consideration? Our views have lately been set forth to the authorities who preside over these matters, but perhaps I may be allowed to add a few words in the hope of throwing some light, however faint, upon it. Two points, at least, are clear; we must have nurses, and at present they are not forthcoming. And why is this? One obvious reason is that we have to compete with a large and ever-growing demand, and we offer far less inducements than any other branch of the nursing service as to position, comfort, and remuneration, and can we expect any other result than being left behind in the race? But why cannot all these impediments be done away with, and a "State" service be rendered as attractive as any other for women desiring to enter the profession of nursing? The very name "workhouse" has a deterrent effect; they might be called "poor-houses," as they used to be; the service would be that of, and under, the Poor Law, and there is no real reason why we should not compete with other branches of the work. There is no reluctance nor hesitation in entering on training in workhouse infirmaries, but by far the majority, on its completion, turn their thoughts and their steps elsewhere, and enter upon some of the many competing branches of the work, which are only too ready to receive them. Now, the one problem before us seems to be, to endeavour to overcome this tendency to leave the service in which they have been trained, and to offer iuducements to remain in the service of the Poor Law. With this one all-important object in view, may I make one or two suggestions which are not now urged for the first time? Without creating a separate nursing department, or a sub-committee of the Local Government Board (if that is impossible), why should not some friendly arrangement or agreement be arrived at between it and the several Boards of Guardians who possess infirmaries large enough, by which they should give free training to a certain number of women over and above those required for the staff of their own infirmary? These to be nominated or recommended to workhouse infirmaries by a committee of experts appointed by the central board. The free training would doubtless attract many, and the inducements which would be given by the prestige of belonging to a State department would attract some. There should be, of course, a fixed term of service under the Poor Law, with the promise of a pension at the end. Some gratuity might possibly be given at the conclusion of the agreement. I ask again, why should not the central board and the several Boards of Guardians co-operate in helping forward a work so sorely needed and earnestly desired by both, but which, it seems, neither can undertake alone; for it is quite clear that the latter cannot call upon the rates for the means of training a sufficient supply of nurses for their needs. No suggestion will probably ever meet the needs of the small country workhouses, which could seldom attract the best nurses, and the only possible remedy I can propose is, to gather the limited numbers of the sick from several workhouses into central or district buildings, many of which are at present half, or more than half, empty, as has already been done with schools and other asylums. It is, of course, frankly allowed by all that many changes are necessary in the position and treatment of nurses in all Poor Law work, but this has been already so often suggested as the first step required, that it need not be dwelt upon here. At the same time these changes must form the first step in the long desired and urged progress of reform, if any is to be carried out.

### To Nurses.

We invite contributions from any of our readers, and shall be glad to pay for "Notes on News from the Nursing World," or for articles describing nursing experiences, or dealing with any nursing question from an original point of view. The minimum payment for contributions is 5s., but we welcome interesting contributions of a column, or a page, in length. It may be added that notices of entertainments, presentations, and deaths are not paid for, but, of course, we are always glad to receive them. All rejected manuscripts are returned in due course, and all payments for manuscripts used are made as early as possible after the beginning of each quarter.

### For Reading to the Sick.

ST. MICHAEL AND ALL ANGELS.

"To Thee all Angels cry aloud, the heavens and all the powers therein."—*Te Deum.*

"He shall give His Angels charge over thee to keep thee in all thy ways."—*Ps.* xci. 2.

Stray notes of everlasting harmonies,
Forms unsubstantial, and memorials dim,—
Faint flittings of the gales of Paradise,
And echoes from the songs of Cherubim;
To aid us are they wafted from on high;—
Sure we may deem that Angels linger by!

But most they love the simple virgin-heart
That humbly strives, advancing day by day,
In their eternal choirs to learn its part;
Till from these troubled waters borne away,
It finds sure refuge in the realms of rest,
And swells the deathless anthems of the blest.

*Lyra Sanctorum.*

No Christian is so humble, but he has Angels to attend on him, if he lives by faith and love. Though they are so great, so glorious, so pure, so wonderful, that the very sight of them (if we were allowed to see them) would strike us to the earth, as it did the prophet Daniel, holy and righteous as he was; yet they are our "fellow-servants" and our fellow-workers, and they carefully watch over and defend even the humblest of us, if we be Christ's. . . . Persons commonly speak as if the other world did not exist now, but would after death. No, it exists now, though we see it not. It is among us and around us. Jacob was shown this in his dream. Angels were all about him, though he knew it not. And what Jacob saw in his sleep, that Elisha's servant saw as if with his eyes; and the shepherds at the time of the Nativity, not only saw, but heard. They heard the voices of those blessed spirits who praise God day and night, and whom we, in our lower state of being, are allowed to copy and to assist.

We are, then, in a world of spirits, as well as in a world of sense, and we hold communion with it, and take part in it, though we are not conscious of doing so. . . . The world of spirits, though unseen, is present: present not future, not distant. It is not above the sky, it is not beyond the grave; it is now and here, the kingdom of God is among us. Of this St. Paul speaks: "We look not at the things which are seen, but at the things which are not seen; for the things which are seen are temporal, but the things which are not seen are eternal."—*T. H. Newman.*

Since to Thy little ones is given such grace,
That they who nearest stand
Alway to God in Heaven, and see His face,
Go forth at His command,
To wait around our path in weal or woe,
As erst upon our King,
Set Thy baptismal seal upon our brow,
And waft us heavenward with enfolding wing:

Grant, Lord, that when around the expiring world
Our Seraph guardians wait,
While on her death-bed, ere to ruin hurled,
She owns Thee, all too late,
They to their charge may turn, and thankful see
Thy mark upon us still;
Then altogether rise, and reign with Thee,
And all their holy joy o'er contrite hearts fulfil!—*Keble.*

### Notes and Queries.

**The Editor is always willing to answer in this column, without any fee, all reasonable questions, as soon as possible.**
**But the following rules must be carefully observed:—**
**1. Every communication must be accompanied by the name and address of the writer.**
**2. The question must always bear upon nursing, directly or indirectly.**
**If an answer is required by letter a fee of half-a-crown must be enclosed with the note containing the inquiry.**

*Tallerman Treatment.*

(239) I should be greatly obliged if you can tell me where I can obtain the "Tallerman" or "baking" treatment for acute rheumatism?—*S. M.*
Apply the Dowsing Radiant Heat Company, 24 Budge Row, Cannon Street.

*Home for Young Man.*

(240) Could you tell me of a surgical home where a young man, recovering from an operation, could be received for a small fee? The wound is not yet healed.—*S. J. D.*
The Kenilworth Convalescent Home, Kenilworth, appears suitable for the case. Also the Sister Dora Memorial Convalescent Hospital, Milford, Stafford.

*Phthisis.*

(241) Will you kindly tell me if there are any free sanatoria for consumption, or if a nurse could be received in one by paying £1 weekly?—*B.*
A list of institutions appeared in THE HOSPITAL of April 20th.

(242) Will you kindly inform me of the date of issue containing a list of the sanatoria in Great Britain?—*J. B.*
See reply to *B.*

(243) Can you tell me of a hospital for the open-air treatment of phthisis where the fees are less than £2 weekly?—*J. M. A. H.*
See the "Sanatoria for Consumption" published in THE HOSPITAL for April 20th. The open-air treatment is used in the National Hospital for Consumption, Ventnor. The Hospital for Consumption, Brompton, and the North London Hospital for Consumption, Mount Vernon, Hampstead, N.W., and others.

*Queen's Nurse.*

(244) Will you kindly tell me how to become a Queen's Nurse? Where must I write for information?—*Joe.*
To the General Superintendent, Queen Victoria's Jubilee Institute for Nurses, St. Katherine's Precincts, Regent's Park, N.W.

*Greek Hospital, Alexandria.*

(245) Will you kindly tell me if it is nice at the Greek Hospital, Alexandria; and also if the air is pleasant there?—*N. B.*
It is hardly possible for us to answer these questions. The climate of Alexandria is very hot in the summer.

*Massage.*

(246) Is there a place in Manchester where I could learn massage?—*E. L. J.*
Write to the Secretary of the Society of Trained Masseuses, 12 Buckingham Street, Strand, W.C., and ask if she can recommend you a teacher.

*Uniform.*

(247) Can you tell me if a certificated masseuse who is not a trained nurse can wear uniform if the patient wishes it?—*S. E. A.*
Anyone can adopt a distinctive dress; but it is as well to select one different from that peculiar to other institutions.

*Paper Pillows.*

(248) Can you tell me how to make paper pillows—such as were supplied to the soldiers in Africa lately? What sort of paper is the best?—*Nurse E. B.*
Newspaper, cut into long narrow strips, is as good as any.

*Chiropody.*

(249) Can you tell me where I could get instruction in chiropody and manicure; and what the fees would be?—*E. N.*
Go to a good chiropodist and manicurist and ask what he or she would charge to teach you.

*Training in Child Nursing.*

(250) I am constantly seeing requests for instruction in the nursing of infants in the NURSING MIRROR. I am now advertising in THE HOSPITAL, and enclose references; may I ask you to give my name to inquirers?—*M. R. B. N. A.*
Your letter has been filed for future reference: but it is against our rules to recommend private establishments.

**Standard Books of Reference.**

**"The Nursing Profession: How and Where to Train." 2s. net; post free 2s. 4d.**
**"Burdett's Official Nursing Directory." 3s. net.; post free, 3s. 4d.**
**"Burdett's Hospitals and Charities." 5s.**
**"The Nurses' Dictionary of Medical Terms." 2s.**
**"Burdett's Series of Nursing Text-Books." 1s. each.**
**"A Handbook for Nurses." (Illustrated.) 5s.**
**"Nursing: Its Theory and Practice." New Edition. 3s. 6d.**
**"Helps in Sickness and to Health." Fifteenth Thousand. 5s.**
**"The Physiological Feeding of Infants." 1s.**
**"The Physiological Nursery Chart." 1s.; post free, 1s. 3d.**
**"Hospital Expenditure: The Commissariat." 2s. 6d.**
**All these are published by THE SCIENTIFIC PRESS, Ltd., and may be obtained through any bookseller or direct from the publishers, 28 and 29 Southampton Street, London, W.C.**

### Travel Notes.

By Our Travelling Correspondent.

### LXXXII.—THE CATHEDRAL AND TOWN OF WELLS.

### Old Houses in Glastonbury.

I had not space last week to tell you of several extremely interesting old houses in the town itself. First and foremost I should place the old Pilgrim's Inn, now called the "George Inn." This fine house, built about the middle of the fifteenth century, was used as a kind of overflow hostelry at the time of great pilgrimages, when the monastic buildings proved insufficient to accommodate the vast crowds that flocked to the chapel of St. Joseph of Arimathea. The hostess kindly allowed me to explore the rooms at my will and study the old dining hall, quaint spiral staircase, and minute windows in the best guest chambers, where some of the original glass, dim and rich still, remains in these very inadequate openings for light and ventilation. Further up the street, on the same side, is a house which goes by the name of "The Tribunal," and was the old court-house where petty cases were heard. The carving on the outside is very rich and in good preservation, but the interior contains little of interest. Next door to the museum stands the "Red Lion" with an opening under a vaulted roof; this leads to a handsome stone gateway, the entrance to the women's almshouses built and endowed by Abbot Beere in 1512.

### The Cathedral Village.

I am told that a scoffer applied this term to the ancient city of Wells, and certainly it seems well placed, for the nice old town is very small and very, very quiet, and appears as an appendage only to the beautiful cathedral and the stately bishop's palace.

It takes its name from the springs which rise in the bishop's garden, and which are popularly supposed to be of unknown depth. The exquisite reflection of the cathedral with its beautiful chapter-house in the pool over the springs, is a sight worth going many miles to see.

Two splendid gates, one called the "Gatehouse" or "Palace Eye," the other "Penniless Porch," lead from the market place into the cathedral precincts.

The greater part of the cathedral, which is dedicated to St. Andrew, was built in the thirteenth century. The west front is exceptionally fine and the effect on entering the nave very grand, though personally I prefer the entrance on the south side from the cloisters, where one comes in under the central tower into the square formed by the four inverted arches, which were placed there very early in the fourteenth century to support the tower, which began to settle very soon after its erection.

These arches cannot be called beautiful, but they are, I believe, unique, and are interesting when one knows why they were built in this curious form. In the north transept is a singular old clock brought from Glastonbury. An ugly little man of ungainly and bloated aspect strikes the quarters with his heels on a bell placed high above the arches, and at the hour various horsemen come out on a platform above the clock and race round on horseback, representing the mediæval pastime of "tilting at the ring." The north porch is very rich in sculpture, and some of it is decidedly quaint. There is a fine yesse window over the high altar, most rich in colour, but, alas! there are also some terrible modern atrocities in that line which, as one of the enlightened vergers regretfully remarked, "would disgrace a travelling caravan."

### The Lady Chapel—Crypt and Chapter House.

Being admitted into the ambulatory, one is allowed to wander at will, and not compelled to go round with a herd at the heels of a guide. The choir disappointed me rather, because it is "restored," but the lady chapel, with its beautiful old windows is very soul satisfying, and as for the crypt, it is perfection, quite "unrestored," I am thankful to say, dim, dark, and cool. One enters through a magnificent oaken door, enriched with hammered iron work, opening from the ambulatory. Strictly speaking, this fine room is not a crypt, but an "undercroft" beneath the chapter-house. It is very little sunk beneath the surrounding ground, for the near neighbourhood of the springs would prevent the construction of a real crypt. Entering a second massive door one sees on the right above one's head a curious stone lantern which must once have lighted the faithful at their devotions. Just inside this second door is a very singular piece of sculpture, which I suppose must be a piscina. Immediately over the little opening is the figure of a hound gnawing a bone.

The thing that delighted me above all others in the cathedral is the beautiful approach by a graceful curving flight of steps to the chapter-house which, once attained, does not differ much from other structures of the same kind but the approach is lovely. About two-thirds of the way up a curve to the right reveals the chapter-house, with cross lights and shadows which make a dream of beauty among the clustered pillars at the entrance. By continuing up the steps you arrive at a covered way over the "chain gate" from the vicar's close, so as to give a shelter to the reverend gentlemen when in olden days they were coming at all hours of the night to services in the sanctuary.

### The Bishop's Palace and Garden.

Having entered the cathedral precincts by the "palace eye" one comes on the right upon the extensive moat which nce formed a convenient fortification for the bishop against warlike attacks from the powerful monks of Glastonbury. This is crossed by a grand tower gate and drawbridge, still in perfect preservation. A very intelligent porter (an old soldier) acts as guide and shows considerable interest in, and knowledge of, his subject.

Parts of the palace date from 1242, and much remains untouched and as strong as ever. One of the most interesting parts is the kitchen with a solid square tower at the end and a perfectly lovely oriel window; in one of the rooms close to this tower a former bishop and his wife were killed by the fall of a chimney-stack which had been struck by lightning. A charming raised walk on the ramparts is called Bishop Ken's walk, and here he composed his morning and evening hymns.

One can get lodgings in a delightful place called the vicar's close, of which I should like to obtain more accurate information. Every chimney seems to have a different coat of arms carved on it, and at the head of the close is a tiny chapel, a perfect gem of the fifteenth century. I had hoped to speak of Sedgemoor and Bridgewater this week, but I have filled my allotted space and must wait to finish my account of this interesting neighbourhood a little longer.

### TRAVEL NOTES AND QUERIES.

Rules in Regard to Correspondence for this Section.—All questioners must use a pseudonym for publication, but the communication must also bear the writer's own name and address as well, which will be regarded as confidential. All such communications to be addressed "Travel Editor, 'Nursing Mirror,' 28 Southampton Street, Strand." No charge will be made for inserting and answering questions in the inquiry column, and all will be answered in rotation as space permits. If an answer by letter is required, a stamped and addressed envelope must be enclosed, together with 2s. 6d., which fee will be devoted to the objects of the "Hospital" Convalescent Fund. Any inquiries reaching the office after Monday cannot be answered in "The Mirror" of the current week.

Paying Guests (Tyneside).—If you will send me a stamped and addressed envelope I will give you the address at Meare. Being a private one I must not publish it in the paper.

The Scilly Isles (Nurse P.).—You give me no pseudonym. I hope this will catch your eye. Expenses of journey, third single, to Penzance, 25s. 3d.; return second, 55s. 4d. Thence a short drive to Land's End by coach, and then by steamer. Fare, second class, 5s. St. Mary's is the principal place in the islands, and is nine or ten miles round. Expense of living fairly moderate, but accommodation not very good in lodgings, and the hotels dear, because of lack of competition and uncertainty of custom. The doubt as to going and coming, the mails being dependent upon weather, makes a short visit undesirable, but if you stayed there some time, this would not matter. After St. Mary's, Tresco and St. Martin are the largest islands, after these St. Agnes-Samson, &c. . . . The attractions are the mild climate, primitive customs, and the interest of the flower growing industry. For good walkers there is much to be seen in coast scenery. If you think of going, I will tell you the usual days the boats leave and return.

